# Supplementary figures and images for: EFNet: estimation of left ventricular ejection fraction from cardiac ultrasound videos using deep learning (part 2 of 2)
Source: PeerJ Comput Sci. 2025 Jan 21;11:e2506. doi: 10.7717/peerj-cs.2506 (PMC11784862; doi:10.7717/peerj-cs.2506)

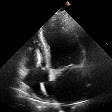

Supplement: Supplemental Information 1 [file peerj-cs-11-2506-s001.zip › EFNet Files/esed_data/esed_data/train/0X100E491B3CD58DE2/frame73.png]

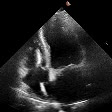

Supplement: Supplemental Information 1 [file peerj-cs-11-2506-s001.zip › EFNet Files/esed_data/esed_data/train/0X100E491B3CD58DE2/frame74.png]

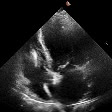

Supplement: Supplemental Information 1 [file peerj-cs-11-2506-s001.zip › EFNet Files/esed_data/esed_data/train/0X100E491B3CD58DE2/v2ed.png]

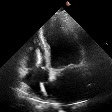

Supplement: Supplemental Information 1 [file peerj-cs-11-2506-s001.zip › EFNet Files/esed_data/esed_data/train/0X100E491B3CD58DE2/v2es.png]

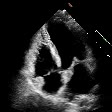

Supplement: Supplemental Information 1 [file peerj-cs-11-2506-s001.zip › EFNet Files/esed_data/esed_data/train/0X100F044876B98F90/frame57.png]

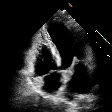

Supplement: Supplemental Information 1 [file peerj-cs-11-2506-s001.zip › EFNet Files/esed_data/esed_data/train/0X100F044876B98F90/frame58.png]

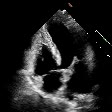

Supplement: Supplemental Information 1 [file peerj-cs-11-2506-s001.zip › EFNet Files/esed_data/esed_data/train/0X100F044876B98F90/frame59.png]

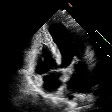

Supplement: Supplemental Information 1 [file peerj-cs-11-2506-s001.zip › EFNet Files/esed_data/esed_data/train/0X100F044876B98F90/frame60.png]

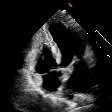

Supplement: Supplemental Information 1 [file peerj-cs-11-2506-s001.zip › EFNet Files/esed_data/esed_data/train/0X100F044876B98F90/frame61.png]

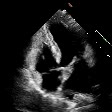

Supplement: Supplemental Information 1 [file peerj-cs-11-2506-s001.zip › EFNet Files/esed_data/esed_data/train/0X100F044876B98F90/frame62.png]

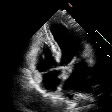

Supplement: Supplemental Information 1 [file peerj-cs-11-2506-s001.zip › EFNet Files/esed_data/esed_data/train/0X100F044876B98F90/frame63.png]

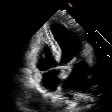

Supplement: Supplemental Information 1 [file peerj-cs-11-2506-s001.zip › EFNet Files/esed_data/esed_data/train/0X100F044876B98F90/frame64.png]

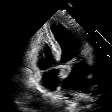

Supplement: Supplemental Information 1 [file peerj-cs-11-2506-s001.zip › EFNet Files/esed_data/esed_data/train/0X100F044876B98F90/frame65.png]

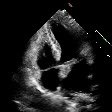

Supplement: Supplemental Information 1 [file peerj-cs-11-2506-s001.zip › EFNet Files/esed_data/esed_data/train/0X100F044876B98F90/frame66.png]

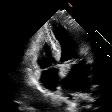

Supplement: Supplemental Information 1 [file peerj-cs-11-2506-s001.zip › EFNet Files/esed_data/esed_data/train/0X100F044876B98F90/frame67.png]

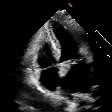

Supplement: Supplemental Information 1 [file peerj-cs-11-2506-s001.zip › EFNet Files/esed_data/esed_data/train/0X100F044876B98F90/frame68.png]

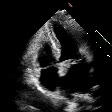

Supplement: Supplemental Information 1 [file peerj-cs-11-2506-s001.zip › EFNet Files/esed_data/esed_data/train/0X100F044876B98F90/frame69.png]

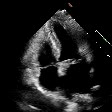

Supplement: Supplemental Information 1 [file peerj-cs-11-2506-s001.zip › EFNet Files/esed_data/esed_data/train/0X100F044876B98F90/frame70.png]

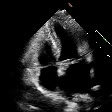

Supplement: Supplemental Information 1 [file peerj-cs-11-2506-s001.zip › EFNet Files/esed_data/esed_data/train/0X100F044876B98F90/frame71.png]

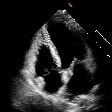

Supplement: Supplemental Information 1 [file peerj-cs-11-2506-s001.zip › EFNet Files/esed_data/esed_data/train/0X100F044876B98F90/v3ed.png]

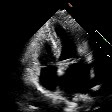

Supplement: Supplemental Information 1 [file peerj-cs-11-2506-s001.zip › EFNet Files/esed_data/esed_data/train/0X100F044876B98F90/v3es.png]

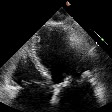

Supplement: Supplemental Information 1 [file peerj-cs-11-2506-s001.zip › EFNet Files/esed_data/esed_data/train/0X101026B90DAE7E95/frame45.png]

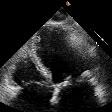

Supplement: Supplemental Information 1 [file peerj-cs-11-2506-s001.zip › EFNet Files/esed_data/esed_data/train/0X101026B90DAE7E95/frame46.png]

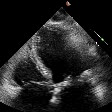

Supplement: Supplemental Information 1 [file peerj-cs-11-2506-s001.zip › EFNet Files/esed_data/esed_data/train/0X101026B90DAE7E95/frame47.png]

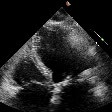

Supplement: Supplemental Information 1 [file peerj-cs-11-2506-s001.zip › EFNet Files/esed_data/esed_data/train/0X101026B90DAE7E95/frame48.png]

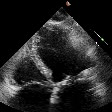

Supplement: Supplemental Information 1 [file peerj-cs-11-2506-s001.zip › EFNet Files/esed_data/esed_data/train/0X101026B90DAE7E95/frame49.png]

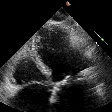

Supplement: Supplemental Information 1 [file peerj-cs-11-2506-s001.zip › EFNet Files/esed_data/esed_data/train/0X101026B90DAE7E95/frame50.png]

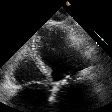

Supplement: Supplemental Information 1 [file peerj-cs-11-2506-s001.zip › EFNet Files/esed_data/esed_data/train/0X101026B90DAE7E95/frame51.png]

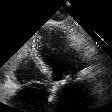

Supplement: Supplemental Information 1 [file peerj-cs-11-2506-s001.zip › EFNet Files/esed_data/esed_data/train/0X101026B90DAE7E95/frame52.png]

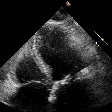

Supplement: Supplemental Information 1 [file peerj-cs-11-2506-s001.zip › EFNet Files/esed_data/esed_data/train/0X101026B90DAE7E95/frame53.png]

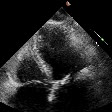

Supplement: Supplemental Information 1 [file peerj-cs-11-2506-s001.zip › EFNet Files/esed_data/esed_data/train/0X101026B90DAE7E95/frame54.png]

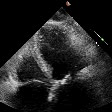

Supplement: Supplemental Information 1 [file peerj-cs-11-2506-s001.zip › EFNet Files/esed_data/esed_data/train/0X101026B90DAE7E95/frame55.png]

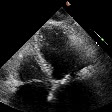

Supplement: Supplemental Information 1 [file peerj-cs-11-2506-s001.zip › EFNet Files/esed_data/esed_data/train/0X101026B90DAE7E95/frame56.png]

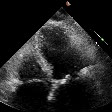

Supplement: Supplemental Information 1 [file peerj-cs-11-2506-s001.zip › EFNet Files/esed_data/esed_data/train/0X101026B90DAE7E95/frame57.png]

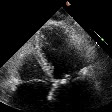

Supplement: Supplemental Information 1 [file peerj-cs-11-2506-s001.zip › EFNet Files/esed_data/esed_data/train/0X101026B90DAE7E95/frame58.png]

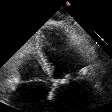

Supplement: Supplemental Information 1 [file peerj-cs-11-2506-s001.zip › EFNet Files/esed_data/esed_data/train/0X101026B90DAE7E95/frame59.png]

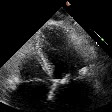

Supplement: Supplemental Information 1 [file peerj-cs-11-2506-s001.zip › EFNet Files/esed_data/esed_data/train/0X101026B90DAE7E95/frame60.png]

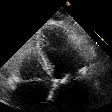

Supplement: Supplemental Information 1 [file peerj-cs-11-2506-s001.zip › EFNet Files/esed_data/esed_data/train/0X101026B90DAE7E95/frame61.png]

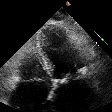

Supplement: Supplemental Information 1 [file peerj-cs-11-2506-s001.zip › EFNet Files/esed_data/esed_data/train/0X101026B90DAE7E95/frame62.png]

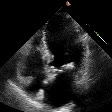

Supplement: Supplemental Information 1 [file peerj-cs-11-2506-s001.zip › EFNet Files/esed_data/esed_data/val/0X100009310A3BD7FC/frame46.png]

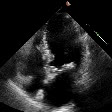

Supplement: Supplemental Information 1 [file peerj-cs-11-2506-s001.zip › EFNet Files/esed_data/esed_data/val/0X100009310A3BD7FC/frame47.png]

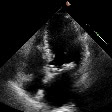

Supplement: Supplemental Information 1 [file peerj-cs-11-2506-s001.zip › EFNet Files/esed_data/esed_data/val/0X100009310A3BD7FC/frame48.png]

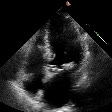

Supplement: Supplemental Information 1 [file peerj-cs-11-2506-s001.zip › EFNet Files/esed_data/esed_data/val/0X100009310A3BD7FC/frame49.png]

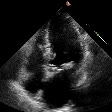

Supplement: Supplemental Information 1 [file peerj-cs-11-2506-s001.zip › EFNet Files/esed_data/esed_data/val/0X100009310A3BD7FC/frame50.png]

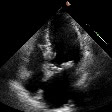

Supplement: Supplemental Information 1 [file peerj-cs-11-2506-s001.zip › EFNet Files/esed_data/esed_data/val/0X100009310A3BD7FC/frame51.png]

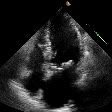

Supplement: Supplemental Information 1 [file peerj-cs-11-2506-s001.zip › EFNet Files/esed_data/esed_data/val/0X100009310A3BD7FC/frame52.png]

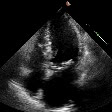

Supplement: Supplemental Information 1 [file peerj-cs-11-2506-s001.zip › EFNet Files/esed_data/esed_data/val/0X100009310A3BD7FC/frame53.png]

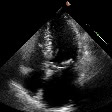

Supplement: Supplemental Information 1 [file peerj-cs-11-2506-s001.zip › EFNet Files/esed_data/esed_data/val/0X100009310A3BD7FC/frame54.png]

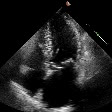

Supplement: Supplemental Information 1 [file peerj-cs-11-2506-s001.zip › EFNet Files/esed_data/esed_data/val/0X100009310A3BD7FC/frame55.png]

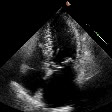

Supplement: Supplemental Information 1 [file peerj-cs-11-2506-s001.zip › EFNet Files/esed_data/esed_data/val/0X100009310A3BD7FC/frame56.png]

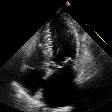

Supplement: Supplemental Information 1 [file peerj-cs-11-2506-s001.zip › EFNet Files/esed_data/esed_data/val/0X100009310A3BD7FC/frame57.png]

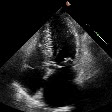

Supplement: Supplemental Information 1 [file peerj-cs-11-2506-s001.zip › EFNet Files/esed_data/esed_data/val/0X100009310A3BD7FC/frame58.png]

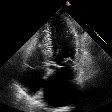

Supplement: Supplemental Information 1 [file peerj-cs-11-2506-s001.zip › EFNet Files/esed_data/esed_data/val/0X100009310A3BD7FC/frame59.png]

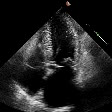

Supplement: Supplemental Information 1 [file peerj-cs-11-2506-s001.zip › EFNet Files/esed_data/esed_data/val/0X100009310A3BD7FC/frame60.png]

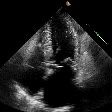

Supplement: Supplemental Information 1 [file peerj-cs-11-2506-s001.zip › EFNet Files/esed_data/esed_data/val/0X100009310A3BD7FC/frame61.png]

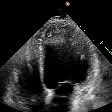

Supplement: Supplemental Information 1 [file peerj-cs-11-2506-s001.zip › EFNet Files/esed_data/esed_data/val/0X10094BA0A028EAC3/frame137.png]

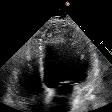

Supplement: Supplemental Information 1 [file peerj-cs-11-2506-s001.zip › EFNet Files/esed_data/esed_data/val/0X10094BA0A028EAC3/frame138.png]

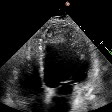

Supplement: Supplemental Information 1 [file peerj-cs-11-2506-s001.zip › EFNet Files/esed_data/esed_data/val/0X10094BA0A028EAC3/frame139.png]

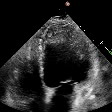

Supplement: Supplemental Information 1 [file peerj-cs-11-2506-s001.zip › EFNet Files/esed_data/esed_data/val/0X10094BA0A028EAC3/frame140.png]

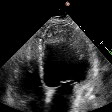

Supplement: Supplemental Information 1 [file peerj-cs-11-2506-s001.zip › EFNet Files/esed_data/esed_data/val/0X10094BA0A028EAC3/frame141.png]

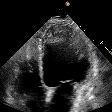

Supplement: Supplemental Information 1 [file peerj-cs-11-2506-s001.zip › EFNet Files/esed_data/esed_data/val/0X10094BA0A028EAC3/frame142.png]

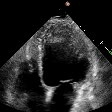

Supplement: Supplemental Information 1 [file peerj-cs-11-2506-s001.zip › EFNet Files/esed_data/esed_data/val/0X10094BA0A028EAC3/frame143.png]

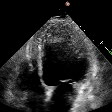

Supplement: Supplemental Information 1 [file peerj-cs-11-2506-s001.zip › EFNet Files/esed_data/esed_data/val/0X10094BA0A028EAC3/frame144.png]

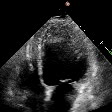

Supplement: Supplemental Information 1 [file peerj-cs-11-2506-s001.zip › EFNet Files/esed_data/esed_data/val/0X10094BA0A028EAC3/frame145.png]

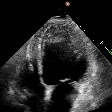

Supplement: Supplemental Information 1 [file peerj-cs-11-2506-s001.zip › EFNet Files/esed_data/esed_data/val/0X10094BA0A028EAC3/frame146.png]

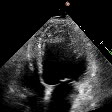

Supplement: Supplemental Information 1 [file peerj-cs-11-2506-s001.zip › EFNet Files/esed_data/esed_data/val/0X10094BA0A028EAC3/frame147.png]

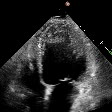

Supplement: Supplemental Information 1 [file peerj-cs-11-2506-s001.zip › EFNet Files/esed_data/esed_data/val/0X10094BA0A028EAC3/frame148.png]

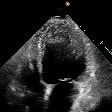

Supplement: Supplemental Information 1 [file peerj-cs-11-2506-s001.zip › EFNet Files/esed_data/esed_data/val/0X10094BA0A028EAC3/frame149.png]

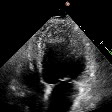

Supplement: Supplemental Information 1 [file peerj-cs-11-2506-s001.zip › EFNet Files/esed_data/esed_data/val/0X10094BA0A028EAC3/frame150.png]

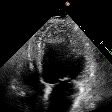

Supplement: Supplemental Information 1 [file peerj-cs-11-2506-s001.zip › EFNet Files/esed_data/esed_data/val/0X10094BA0A028EAC3/frame151.png]

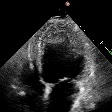

Supplement: Supplemental Information 1 [file peerj-cs-11-2506-s001.zip › EFNet Files/esed_data/esed_data/val/0X10094BA0A028EAC3/frame152.png]

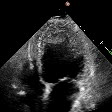

Supplement: Supplemental Information 1 [file peerj-cs-11-2506-s001.zip › EFNet Files/esed_data/esed_data/val/0X10094BA0A028EAC3/frame153.png]

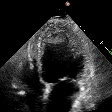

Supplement: Supplemental Information 1 [file peerj-cs-11-2506-s001.zip › EFNet Files/esed_data/esed_data/val/0X10094BA0A028EAC3/frame154.png]

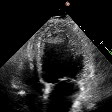

Supplement: Supplemental Information 1 [file peerj-cs-11-2506-s001.zip › EFNet Files/esed_data/esed_data/val/0X10094BA0A028EAC3/frame155.png]

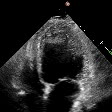

Supplement: Supplemental Information 1 [file peerj-cs-11-2506-s001.zip › EFNet Files/esed_data/esed_data/val/0X10094BA0A028EAC3/frame156.png]
