# Supplementary figures and images for: EFNet: estimation of left ventricular ejection fraction from cardiac ultrasound videos using deep learning (part 1 of 2)
Source: PeerJ Comput Sci. 2025 Jan 21;11:e2506. doi: 10.7717/peerj-cs.2506 (PMC11784862; doi:10.7717/peerj-cs.2506)

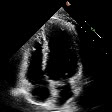

Supplement: Supplemental Information 1 [file peerj-cs-11-2506-s001.zip › EFNet Files/esed_data/esed_data/test/0X100CF05D141FF143/frame132.png]

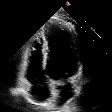

Supplement: Supplemental Information 1 [file peerj-cs-11-2506-s001.zip › EFNet Files/esed_data/esed_data/test/0X100CF05D141FF143/frame133.png]

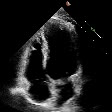

Supplement: Supplemental Information 1 [file peerj-cs-11-2506-s001.zip › EFNet Files/esed_data/esed_data/test/0X100CF05D141FF143/frame134.png]

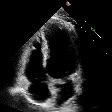

Supplement: Supplemental Information 1 [file peerj-cs-11-2506-s001.zip › EFNet Files/esed_data/esed_data/test/0X100CF05D141FF143/frame135.png]

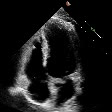

Supplement: Supplemental Information 1 [file peerj-cs-11-2506-s001.zip › EFNet Files/esed_data/esed_data/test/0X100CF05D141FF143/frame136.png]

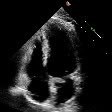

Supplement: Supplemental Information 1 [file peerj-cs-11-2506-s001.zip › EFNet Files/esed_data/esed_data/test/0X100CF05D141FF143/frame137.png]

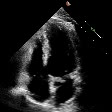

Supplement: Supplemental Information 1 [file peerj-cs-11-2506-s001.zip › EFNet Files/esed_data/esed_data/test/0X100CF05D141FF143/frame138.png]

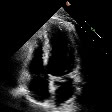

Supplement: Supplemental Information 1 [file peerj-cs-11-2506-s001.zip › EFNet Files/esed_data/esed_data/test/0X100CF05D141FF143/frame139.png]

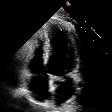

Supplement: Supplemental Information 1 [file peerj-cs-11-2506-s001.zip › EFNet Files/esed_data/esed_data/test/0X100CF05D141FF143/frame140.png]

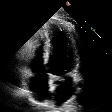

Supplement: Supplemental Information 1 [file peerj-cs-11-2506-s001.zip › EFNet Files/esed_data/esed_data/test/0X100CF05D141FF143/frame141.png]

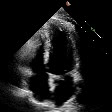

Supplement: Supplemental Information 1 [file peerj-cs-11-2506-s001.zip › EFNet Files/esed_data/esed_data/test/0X100CF05D141FF143/frame142.png]

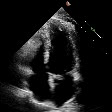

Supplement: Supplemental Information 1 [file peerj-cs-11-2506-s001.zip › EFNet Files/esed_data/esed_data/test/0X100CF05D141FF143/frame143.png]

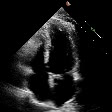

Supplement: Supplemental Information 1 [file peerj-cs-11-2506-s001.zip › EFNet Files/esed_data/esed_data/test/0X100CF05D141FF143/frame144.png]

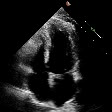

Supplement: Supplemental Information 1 [file peerj-cs-11-2506-s001.zip › EFNet Files/esed_data/esed_data/test/0X100CF05D141FF143/frame145.png]

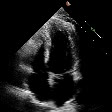

Supplement: Supplemental Information 1 [file peerj-cs-11-2506-s001.zip › EFNet Files/esed_data/esed_data/test/0X100CF05D141FF143/frame146.png]

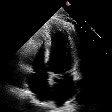

Supplement: Supplemental Information 1 [file peerj-cs-11-2506-s001.zip › EFNet Files/esed_data/esed_data/test/0X100CF05D141FF143/frame147.png]

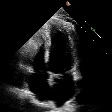

Supplement: Supplemental Information 1 [file peerj-cs-11-2506-s001.zip › EFNet Files/esed_data/esed_data/test/0X100CF05D141FF143/frame148.png]

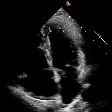

Supplement: Supplemental Information 1 [file peerj-cs-11-2506-s001.zip › EFNet Files/esed_data/esed_data/train/0X1002E8FBACD08477/frame10.png]

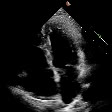

Supplement: Supplemental Information 1 [file peerj-cs-11-2506-s001.zip › EFNet Files/esed_data/esed_data/train/0X1002E8FBACD08477/frame11.png]

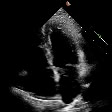

Supplement: Supplemental Information 1 [file peerj-cs-11-2506-s001.zip › EFNet Files/esed_data/esed_data/train/0X1002E8FBACD08477/frame12.png]

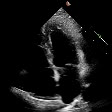

Supplement: Supplemental Information 1 [file peerj-cs-11-2506-s001.zip › EFNet Files/esed_data/esed_data/train/0X1002E8FBACD08477/frame13.png]

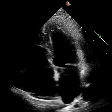

Supplement: Supplemental Information 1 [file peerj-cs-11-2506-s001.zip › EFNet Files/esed_data/esed_data/train/0X1002E8FBACD08477/frame14.png]

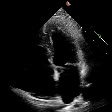

Supplement: Supplemental Information 1 [file peerj-cs-11-2506-s001.zip › EFNet Files/esed_data/esed_data/train/0X1002E8FBACD08477/frame15.png]

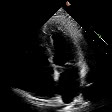

Supplement: Supplemental Information 1 [file peerj-cs-11-2506-s001.zip › EFNet Files/esed_data/esed_data/train/0X1002E8FBACD08477/frame16.png]

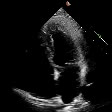

Supplement: Supplemental Information 1 [file peerj-cs-11-2506-s001.zip › EFNet Files/esed_data/esed_data/train/0X1002E8FBACD08477/frame17.png]

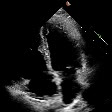

Supplement: Supplemental Information 1 [file peerj-cs-11-2506-s001.zip › EFNet Files/esed_data/esed_data/train/0X1002E8FBACD08477/frame4.png]

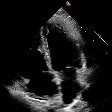

Supplement: Supplemental Information 1 [file peerj-cs-11-2506-s001.zip › EFNet Files/esed_data/esed_data/train/0X1002E8FBACD08477/frame5.png]

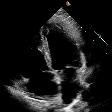

Supplement: Supplemental Information 1 [file peerj-cs-11-2506-s001.zip › EFNet Files/esed_data/esed_data/train/0X1002E8FBACD08477/frame6.png]

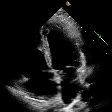

Supplement: Supplemental Information 1 [file peerj-cs-11-2506-s001.zip › EFNet Files/esed_data/esed_data/train/0X1002E8FBACD08477/frame7.png]

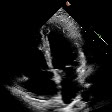

Supplement: Supplemental Information 1 [file peerj-cs-11-2506-s001.zip › EFNet Files/esed_data/esed_data/train/0X1002E8FBACD08477/frame8.png]

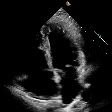

Supplement: Supplemental Information 1 [file peerj-cs-11-2506-s001.zip › EFNet Files/esed_data/esed_data/train/0X1002E8FBACD08477/frame9.png]

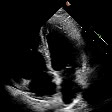

Supplement: Supplemental Information 1 [file peerj-cs-11-2506-s001.zip › EFNet Files/esed_data/esed_data/train/0X1002E8FBACD08477/v4ed.png]

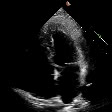

Supplement: Supplemental Information 1 [file peerj-cs-11-2506-s001.zip › EFNet Files/esed_data/esed_data/train/0X1002E8FBACD08477/v4es.png]

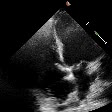

Supplement: Supplemental Information 1 [file peerj-cs-11-2506-s001.zip › EFNet Files/esed_data/esed_data/train/0X1005D03EED19C65B/frame25.png]

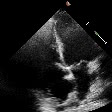

Supplement: Supplemental Information 1 [file peerj-cs-11-2506-s001.zip › EFNet Files/esed_data/esed_data/train/0X1005D03EED19C65B/frame26.png]

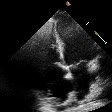

Supplement: Supplemental Information 1 [file peerj-cs-11-2506-s001.zip › EFNet Files/esed_data/esed_data/train/0X1005D03EED19C65B/frame27.png]

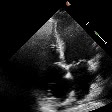

Supplement: Supplemental Information 1 [file peerj-cs-11-2506-s001.zip › EFNet Files/esed_data/esed_data/train/0X1005D03EED19C65B/frame28.png]

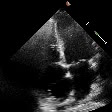

Supplement: Supplemental Information 1 [file peerj-cs-11-2506-s001.zip › EFNet Files/esed_data/esed_data/train/0X1005D03EED19C65B/frame29.png]

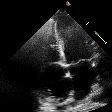

Supplement: Supplemental Information 1 [file peerj-cs-11-2506-s001.zip › EFNet Files/esed_data/esed_data/train/0X1005D03EED19C65B/frame30.png]

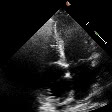

Supplement: Supplemental Information 1 [file peerj-cs-11-2506-s001.zip › EFNet Files/esed_data/esed_data/train/0X1005D03EED19C65B/frame31.png]

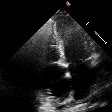

Supplement: Supplemental Information 1 [file peerj-cs-11-2506-s001.zip › EFNet Files/esed_data/esed_data/train/0X1005D03EED19C65B/frame32.png]

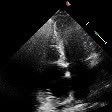

Supplement: Supplemental Information 1 [file peerj-cs-11-2506-s001.zip › EFNet Files/esed_data/esed_data/train/0X1005D03EED19C65B/frame33.png]

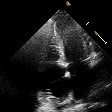

Supplement: Supplemental Information 1 [file peerj-cs-11-2506-s001.zip › EFNet Files/esed_data/esed_data/train/0X1005D03EED19C65B/frame34.png]

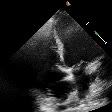

Supplement: Supplemental Information 1 [file peerj-cs-11-2506-s001.zip › EFNet Files/esed_data/esed_data/train/0X1005D03EED19C65B/v5ed.png]

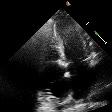

Supplement: Supplemental Information 1 [file peerj-cs-11-2506-s001.zip › EFNet Files/esed_data/esed_data/train/0X1005D03EED19C65B/v5es.png]

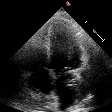

Supplement: Supplemental Information 1 [file peerj-cs-11-2506-s001.zip › EFNet Files/esed_data/esed_data/train/0X10075961BC11C88E/frame100.png]

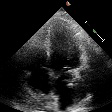

Supplement: Supplemental Information 1 [file peerj-cs-11-2506-s001.zip › EFNet Files/esed_data/esed_data/train/0X10075961BC11C88E/frame101.png]

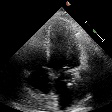

Supplement: Supplemental Information 1 [file peerj-cs-11-2506-s001.zip › EFNet Files/esed_data/esed_data/train/0X10075961BC11C88E/frame102.png]

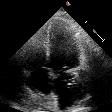

Supplement: Supplemental Information 1 [file peerj-cs-11-2506-s001.zip › EFNet Files/esed_data/esed_data/train/0X10075961BC11C88E/frame103.png]

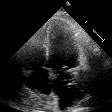

Supplement: Supplemental Information 1 [file peerj-cs-11-2506-s001.zip › EFNet Files/esed_data/esed_data/train/0X10075961BC11C88E/frame104.png]

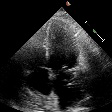

Supplement: Supplemental Information 1 [file peerj-cs-11-2506-s001.zip › EFNet Files/esed_data/esed_data/train/0X10075961BC11C88E/frame105.png]

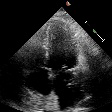

Supplement: Supplemental Information 1 [file peerj-cs-11-2506-s001.zip › EFNet Files/esed_data/esed_data/train/0X10075961BC11C88E/frame106.png]

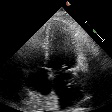

Supplement: Supplemental Information 1 [file peerj-cs-11-2506-s001.zip › EFNet Files/esed_data/esed_data/train/0X10075961BC11C88E/frame107.png]

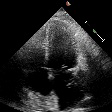

Supplement: Supplemental Information 1 [file peerj-cs-11-2506-s001.zip › EFNet Files/esed_data/esed_data/train/0X10075961BC11C88E/frame108.png]

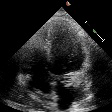

Supplement: Supplemental Information 1 [file peerj-cs-11-2506-s001.zip › EFNet Files/esed_data/esed_data/train/0X10075961BC11C88E/frame91.png]

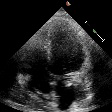

Supplement: Supplemental Information 1 [file peerj-cs-11-2506-s001.zip › EFNet Files/esed_data/esed_data/train/0X10075961BC11C88E/frame92.png]

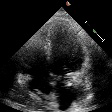

Supplement: Supplemental Information 1 [file peerj-cs-11-2506-s001.zip › EFNet Files/esed_data/esed_data/train/0X10075961BC11C88E/frame93.png]

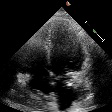

Supplement: Supplemental Information 1 [file peerj-cs-11-2506-s001.zip › EFNet Files/esed_data/esed_data/train/0X10075961BC11C88E/frame94.png]

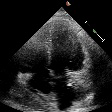

Supplement: Supplemental Information 1 [file peerj-cs-11-2506-s001.zip › EFNet Files/esed_data/esed_data/train/0X10075961BC11C88E/frame95.png]

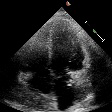

Supplement: Supplemental Information 1 [file peerj-cs-11-2506-s001.zip › EFNet Files/esed_data/esed_data/train/0X10075961BC11C88E/frame96.png]

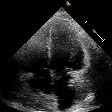

Supplement: Supplemental Information 1 [file peerj-cs-11-2506-s001.zip › EFNet Files/esed_data/esed_data/train/0X10075961BC11C88E/frame97.png]

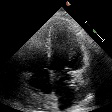

Supplement: Supplemental Information 1 [file peerj-cs-11-2506-s001.zip › EFNet Files/esed_data/esed_data/train/0X10075961BC11C88E/frame98.png]

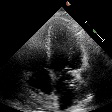

Supplement: Supplemental Information 1 [file peerj-cs-11-2506-s001.zip › EFNet Files/esed_data/esed_data/train/0X10075961BC11C88E/frame99.png]

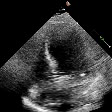

Supplement: Supplemental Information 1 [file peerj-cs-11-2506-s001.zip › EFNet Files/esed_data/esed_data/train/0X100E3B8D3280BEC5/frame26.png]

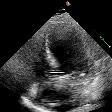

Supplement: Supplemental Information 1 [file peerj-cs-11-2506-s001.zip › EFNet Files/esed_data/esed_data/train/0X100E3B8D3280BEC5/frame27.png]

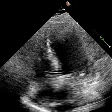

Supplement: Supplemental Information 1 [file peerj-cs-11-2506-s001.zip › EFNet Files/esed_data/esed_data/train/0X100E3B8D3280BEC5/frame28.png]

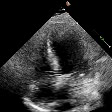

Supplement: Supplemental Information 1 [file peerj-cs-11-2506-s001.zip › EFNet Files/esed_data/esed_data/train/0X100E3B8D3280BEC5/frame29.png]

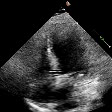

Supplement: Supplemental Information 1 [file peerj-cs-11-2506-s001.zip › EFNet Files/esed_data/esed_data/train/0X100E3B8D3280BEC5/frame30.png]

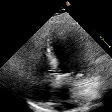

Supplement: Supplemental Information 1 [file peerj-cs-11-2506-s001.zip › EFNet Files/esed_data/esed_data/train/0X100E3B8D3280BEC5/frame31.png]

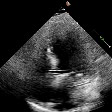

Supplement: Supplemental Information 1 [file peerj-cs-11-2506-s001.zip › EFNet Files/esed_data/esed_data/train/0X100E3B8D3280BEC5/frame32.png]

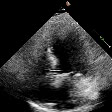

Supplement: Supplemental Information 1 [file peerj-cs-11-2506-s001.zip › EFNet Files/esed_data/esed_data/train/0X100E3B8D3280BEC5/frame33.png]

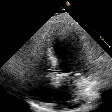

Supplement: Supplemental Information 1 [file peerj-cs-11-2506-s001.zip › EFNet Files/esed_data/esed_data/train/0X100E3B8D3280BEC5/frame34.png]

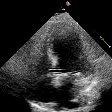

Supplement: Supplemental Information 1 [file peerj-cs-11-2506-s001.zip › EFNet Files/esed_data/esed_data/train/0X100E3B8D3280BEC5/frame35.png]

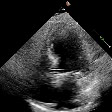

Supplement: Supplemental Information 1 [file peerj-cs-11-2506-s001.zip › EFNet Files/esed_data/esed_data/train/0X100E3B8D3280BEC5/frame36.png]

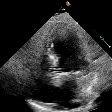

Supplement: Supplemental Information 1 [file peerj-cs-11-2506-s001.zip › EFNet Files/esed_data/esed_data/train/0X100E3B8D3280BEC5/frame37.png]

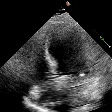

Supplement: Supplemental Information 1 [file peerj-cs-11-2506-s001.zip › EFNet Files/esed_data/esed_data/train/0X100E3B8D3280BEC5/v1ed.png]

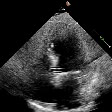

Supplement: Supplemental Information 1 [file peerj-cs-11-2506-s001.zip › EFNet Files/esed_data/esed_data/train/0X100E3B8D3280BEC5/v1es.png]

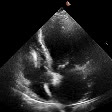

Supplement: Supplemental Information 1 [file peerj-cs-11-2506-s001.zip › EFNet Files/esed_data/esed_data/train/0X100E491B3CD58DE2/frame50.png]

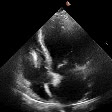

Supplement: Supplemental Information 1 [file peerj-cs-11-2506-s001.zip › EFNet Files/esed_data/esed_data/train/0X100E491B3CD58DE2/frame51.png]

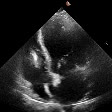

Supplement: Supplemental Information 1 [file peerj-cs-11-2506-s001.zip › EFNet Files/esed_data/esed_data/train/0X100E491B3CD58DE2/frame52.png]

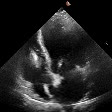

Supplement: Supplemental Information 1 [file peerj-cs-11-2506-s001.zip › EFNet Files/esed_data/esed_data/train/0X100E491B3CD58DE2/frame53.png]

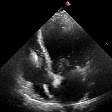

Supplement: Supplemental Information 1 [file peerj-cs-11-2506-s001.zip › EFNet Files/esed_data/esed_data/train/0X100E491B3CD58DE2/frame54.png]

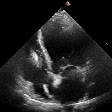

Supplement: Supplemental Information 1 [file peerj-cs-11-2506-s001.zip › EFNet Files/esed_data/esed_data/train/0X100E491B3CD58DE2/frame55.png]

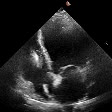

Supplement: Supplemental Information 1 [file peerj-cs-11-2506-s001.zip › EFNet Files/esed_data/esed_data/train/0X100E491B3CD58DE2/frame56.png]

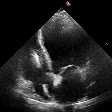

Supplement: Supplemental Information 1 [file peerj-cs-11-2506-s001.zip › EFNet Files/esed_data/esed_data/train/0X100E491B3CD58DE2/frame57.png]

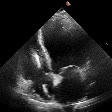

Supplement: Supplemental Information 1 [file peerj-cs-11-2506-s001.zip › EFNet Files/esed_data/esed_data/train/0X100E491B3CD58DE2/frame58.png]

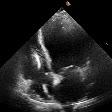

Supplement: Supplemental Information 1 [file peerj-cs-11-2506-s001.zip › EFNet Files/esed_data/esed_data/train/0X100E491B3CD58DE2/frame59.png]

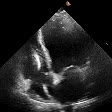

Supplement: Supplemental Information 1 [file peerj-cs-11-2506-s001.zip › EFNet Files/esed_data/esed_data/train/0X100E491B3CD58DE2/frame60.png]

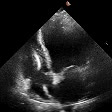

Supplement: Supplemental Information 1 [file peerj-cs-11-2506-s001.zip › EFNet Files/esed_data/esed_data/train/0X100E491B3CD58DE2/frame61.png]

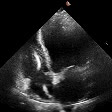

Supplement: Supplemental Information 1 [file peerj-cs-11-2506-s001.zip › EFNet Files/esed_data/esed_data/train/0X100E491B3CD58DE2/frame62.png]

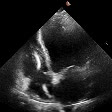

Supplement: Supplemental Information 1 [file peerj-cs-11-2506-s001.zip › EFNet Files/esed_data/esed_data/train/0X100E491B3CD58DE2/frame63.png]

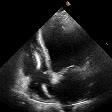

Supplement: Supplemental Information 1 [file peerj-cs-11-2506-s001.zip › EFNet Files/esed_data/esed_data/train/0X100E491B3CD58DE2/frame64.png]

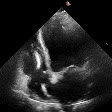

Supplement: Supplemental Information 1 [file peerj-cs-11-2506-s001.zip › EFNet Files/esed_data/esed_data/train/0X100E491B3CD58DE2/frame65.png]

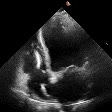

Supplement: Supplemental Information 1 [file peerj-cs-11-2506-s001.zip › EFNet Files/esed_data/esed_data/train/0X100E491B3CD58DE2/frame66.png]

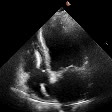

Supplement: Supplemental Information 1 [file peerj-cs-11-2506-s001.zip › EFNet Files/esed_data/esed_data/train/0X100E491B3CD58DE2/frame67.png]

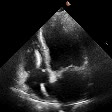

Supplement: Supplemental Information 1 [file peerj-cs-11-2506-s001.zip › EFNet Files/esed_data/esed_data/train/0X100E491B3CD58DE2/frame68.png]

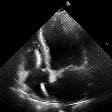

Supplement: Supplemental Information 1 [file peerj-cs-11-2506-s001.zip › EFNet Files/esed_data/esed_data/train/0X100E491B3CD58DE2/frame69.png]

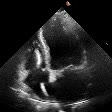

Supplement: Supplemental Information 1 [file peerj-cs-11-2506-s001.zip › EFNet Files/esed_data/esed_data/train/0X100E491B3CD58DE2/frame70.png]

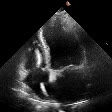

Supplement: Supplemental Information 1 [file peerj-cs-11-2506-s001.zip › EFNet Files/esed_data/esed_data/train/0X100E491B3CD58DE2/frame71.png]

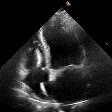

Supplement: Supplemental Information 1 [file peerj-cs-11-2506-s001.zip › EFNet Files/esed_data/esed_data/train/0X100E491B3CD58DE2/frame72.png]
